# Differentially Expressed Genes in Resistant and Susceptible Common Bean (*Phaseolus vulgaris* L.) Genotypes in Response to *Fusarium oxysporum* f. sp. *phaseoli*

**DOI:** 10.1371/journal.pone.0127698

**Published:** 2015-06-01

**Authors:** Renfeng Xue, Jing Wu, Zhendong Zhu, Lanfen Wang, Xiaoming Wang, Shumin Wang, Matthew W. Blair

**Affiliations:** 1 Crop Research Institute, Liaoning Academy of Agricultural Sciences, Shenyang, People’s Republic of China; 2 National Key Facility for Crop Gene Resources and Genetic Improvement, Institute of Crop Science, Chinese Academy of Agricultural Sciences, Beijing, People’s Republic of China; 3 Department of Agricultural and Environmental Sciences, Tennessee State University, Nashville, Tennessee, United States of America; University of California Davis, UNITED STATES

## Abstract

Fusarium wilt of common bean (*Phaseolus vulgaris* L.), caused by *Fusarium oxysporum* Schlechtend.:Fr. f.sp. *phaseoli (Fop)*, is one of the most important diseases of common beans worldwide. Few natural sources of resistance to *Fop* exist and provide only moderate or partial levels of protection. Despite the economic importance of the disease across multiple crops, only a few of *Fop* induced genes have been analyzed in legumes. Therefore, our goal was to identify transcriptionally regulated genes during an incompatible interaction between common bean and the *Fop* pathogen using the cDNA amplified fragment length polymorphism (cDNA-AFLP) technique. We generated a total of 8,730 transcript-derived fragments (TDFs) with 768 primer pairs based on the comparison of a moderately resistant and a susceptible genotype. In total, 423 TDFs (4.9%) displayed altered expression patterns after inoculation with *Fop* inoculum. We obtained full amplicon sequences for 122 selected TDFs, of which 98 were identified as annotated known genes in different functional categories based on their putative functions, 10 were predicted but non-annotated genes and 14 were not homologous to any known genes. The 98 TDFs encoding genes of known putative function were classified as related to metabolism (22), signal transduction (21), protein synthesis and processing (20), development and cytoskeletal organization (12), transport of proteins (7), gene expression and RNA metabolism (4), redox reactions (4), defense and stress responses (3), energy metabolism (3), and hormone responses (2). Based on the analyses of homology, 19 TDFs from different functional categories were chosen for expression analysis using quantitative RT-PCR. The genes found to be important here were implicated at various steps of pathogen infection and will allow a better understanding of the mechanisms of defense and resistance to *Fop* and similar pathogens. The differential response genes discovered here could also be used as molecular markers in association mapping or QTL analysis.

## Introduction

*Fusarium oxysporum* spp. species are ubiquitous fungal pathogens causing Fusarium wilt diseases in a broad range of economically important crop species such as cotton, banana, tomato, legumes, and commercial flowers. Fusarium wilt was first identified on common bean (*Phaseolus vulgaris* L.) in the USA in 1929[1] and since then this pathogen species has been classified as *Fusarium oxysporum* f. sp. *phaseoli* (abbreviated *Fop*). The mode of infection of this soil-borne pathogen is penetration of plant root tissues; and subsequent colonization of the vascular tissue of roots, stem or the whole plant causing phloem blockage, internal stem discoloration and total plant wilt. The main symptoms of beans infected with pathogenic isolates of *Fop* are severe developmental alterations such as stunting and complete wilting, along with extensive chlorosis and necrosis on the leaves and eventual plant death.

Fusarium wilt can have a severe economic impact on common bean fields from seedling up to pre-harvest stages[2]. The pathogen has been detected in most of the bean-growing regions in Africa, East Asia, Europe, Latin America, and the western United States[3]. In China, Fusarium wilt has caused significant economic losses in common bean production due to increased land pressure to grow row crops in monoculture. Especially severe Fusarium wilt epedemics occur in Heilongjiang, a northeast province with limited rotations of corn, soybeans and common beans. The disease is also important in other bean-growing regions of China where common bean fields follow vegetables. Fusarium wilt is also a problem in many other intensively cropped areas of the world, especially as high rainfall and heavy rains become more frequent early in the season with climate change and weather variability. High moisture, excessive irrigation or poorly drained fields and a lack of rotation encourage the disease.

The host-pathogen interactions of crops and Fusarium wilts have been well characterized in only a few *Fusarium oxysporum*-plant pathosystems, including *Musa paradisiaca*[4], *Cucumis melo*[5], *Cicer arietinum*[6,7], *Gossypium hirsutum*[8] and *Lycopersicon esculentum*[9], each with their respective *Fusarium* pathogen, but not so much in Fop interaction with common bean. A few studies of Fusarium wilt infection have been conducted with the model species *Arabidopsis thaliana*[10,11] and *Medicago truncatula*[12]. A variety of defense mechanisms in plant-microbe interactions have been observed for *F*. *oxysporum*, including wound responses and hypersensitive reactions as well as many gene expression and metabolic changes.

At a metabolic level, changes include those in the activity of sugar metabolism genes, such as sucrose synthase, invertase and β-amylase[6]. These reactions are important as they affect sugar concentration in the phloem and sugars serve a dual function as nutrient and as a signal of the disease[13]. The redox status of the intracellular (symplastic) and extracellular (apoplastic) spaces also change with Fusarium wilt infection[14]. In addition, constitutive enzymatic responses to *Fusarium* infection appear to be important with changes in glutathione S-transferases (GST), peroxidases (POX) and phenylalanine ammonia lyase (PAL) enzyme levels and activities being significant upon pathogen attack[15]. Changes also occur in the types and levels of cell wall proteins, proteinase inhibitors, hydrolytic enzymes and pathogenesis-related (PR) proteins and phytoalexin biosynthetic enzymes also appear to play important roles in *Fusarium* wilt defense[15,16].

As most of these defense responses can be monitored at the transcriptional level, a broad genetic screening can provide insights into the type of defense mechanism involved in the Fusarium wilt disease reacton and *Fop*-common bean plant pathosystem. The cDNA amplified fragment length polymorphism (cDNA-AFLP) technique, often called differential display based on its use of observable gel polymorphisms, remains a valuable, low cost, effective, and reliable tool for the study of disease responsive genes at both expression and transcriptional levels. This is true because, single genes and multigene families that are upregulated by disease infection and potentially involved in disease resistance can be uncovered by differential display reactions[17].

Differential display is useful at generating transcript-derived fragment (TDF) libraries from specific treatments, genotypes and disease conditions during varying stages of the infection process and disease reaction in the plant. TDF libraries have been used extensively to characterize the differentially expressed genes at different levels of disease development in order to analyze their function and correlation with disease resistance having the advantage of producing cloned fragments useful for further experiments[18]. As a result, morphological, molecular and anatomical studies have then been carried out to evaluate whether differential display gene expression within specific host tissues is correlated with the onset as well as the progression of the pathogen in the plant[19]. In this sense most differential display studies are more detailed than next generation sequencing (NGS) analysis of plant mRNAs during disease infection, although transcriptome NGS techniques can be a follow up step to differential display analysis and have their own advantages to discovery of many disease induced genes at once, usually through a bioinformatics approach.

The main objective of this study was to determine which common bean genes were transcriptionally regulated in response to *Fop* infection using the cDNA-AFLP and differential display techniques. Quantitative real-time polymerase chain reaction (qRT-PCR) analysis was then used to validate the expression patterns of TDFs of interest. To our knowledge this is the first research to evaluate *Fop*-infected common bean for a large number of TDF derived genes which were activated or suppressed during the interaction between host and pathogen. We discuss a large number of genes which were identified as potential defense-related genes from the early signaling pathogen-response pathway. A better understanding of the basis of resistance in the *F*. *oxysporum* common bean pathosystem will aid in the development of alternative management strategies for Fusarium wilt in this crop. One challenge molecular breeding work faces with Fusarium wilt is the lack of resistant genotypes. In this study we address this with a source of resistance to local isolates of Fop that previously had not been studied.

## Materials and Methods

### Plant materials and fungal inoculation

Seed of two different genotypes: BRB130 (Fusarium wilt susceptible) and CAAS260205 (Fusarium wilt resistant) were obtained from the Institute of Crop Sciences of the Chinese Academy of Agricultural Sciences (CAAS) in Beijing, China[20]. Seeds were both sown in 15-cm diameter pots filled with sterile vermiculite and clay at a volumetric ratio of 3:1. The seedlings were allowed to grow in natural greenhouse conditions at 22 to 28°C and 35 to 40% humidity. At this point non-inoculated control plants were separately labeled from plants to be infected. Ten-day-old seedlings were used for the infection approximately at the fully expanded unifoliolate leaf stage. Plants were infected by an aggressive *F*. *oxysporum* f. sp. *phaseoli* isolate, FOP-DM01 using a previous described method[20]. The control plants were transferred to non-inoculated mixture and provided the same growth conditions along with adequate watering after transplanting. The three plants for each treatment and each time point were fertilized every week with a liquid fertilizer (1% of 17-17-17 of elements N-P-K, Qingdao East Chemical Co., Ltd, 100 ml per pot) and used for cDNA-AFLP experiment. The plants were grown in a greenhouse maintained at approximately 22–28°C. All treatments were grown in the same greenhouse with daylight plus 14 h of supplemental lighting (long day conditions).

### Assay of defense-related enzymes and factors

The activity of two well-known defense enzymes, PAL and POX, were assayed following the method of Xue et al.[21] with some modifications. Enzymatic activity of PAL was determined by the production of cinnamate, measured by the absorbance change at 290 nm with a UV-160 spectrophotometer (Puxi Corp., Beijing, China). The blank was prepared with a mixture containing L-phenylalanine with an equal volume of sodium borate buffer.

POX activity assays were started by adding 100 μl of enzyme extracts, and measured with the absorbance of 470 nm recorded for 5 min at 25°C. The concentration of total protein was estimated by the Bradford method[22]. One unit of the enzyme activity was defined as the amount of enzyme converting 1 μmol of substrate to the production per min. The enzyme activity was expressed as nano ketals per mg of total soluble proteins.

In addition, two factors related to defense response including total hydrogen peroxide (H_2_O_2_) content and superoxide anion (O_2_^-^) production were also measured according to Xue et al.[21] with the following modifications. For the H_2_O_2_ assay, the reaction mixture was centrifuged and the absorbance of the supernatant was measured at 480 nm. H_2_O_2_ content was quantified using a standard curve prepared with plant H_2_O_2_. For the O_2_^-^ assay, the absorbance of the reaction mixture was measured at 530 nm. A standard curve with nitrogen dioxide radical (NO_2_^−^) was used to calculate the production of O_2_^-^. For each of the assays listed above, three replicates were used per sample. The average of each reaction was estimated and used in graphs and figures along with standard deviations calculated with MicroSoft Excel.

### Microscopy

The histology of common bean roots infected by the pathogen were investigated as described previously using scanning electron microscopy(SEM)[7] and transmission electron microscopy (TEM)[23]. Root tissue samples from infected BRB130 plants were collected at 4, 8, 12 days after inoculation, and samples from infected CAAS260205 were collected at 12 and 21 days after inoculation. In SEM, 2 cm long sections of root were excised from the root hair region using a sharp razor blade from all samples at each time point and fixed using 3% glutaraldehyde in 1×PBS (pH 7.2) at 4°C overnight. These sections were washed in 1×PBS buffer three times for 10 min each. The samples were post fixed with 1% (w/v) osmium tetroxide in the same buffer at 4°C for 2 h and washed briefly with distilled water. The samples for analysis were then dehydrated in a graded ethanol series (sequential concentrations of 30, 50, 70, 80, 90 and 100% for 10 min each) at room temperature. The samples were further treated with isoamyl acetate in the same graded fashion (sequential concentrations of 30, 50, 70, 80, 90 and 100% for 10 min each) and dried to a critical point with CO_2_ as the transitional environment. Samples were then mounted on metal slides (10 mm in width) using two-sided adhesive carbon tape. These samples were then coated under an argon atmosphere with a thin layer (approx. 30 nm in thickness) of gold using a spatter coater under accelerating voltage of 20 kV.

In TEM, the root fragments (≈1 mm^3^) were fixed by immersion in 3% (v/v) glutaraldehyde in 0.1 M (pH 7.2) cacodylate buffer for 12 h at 25°C. After washing in this buffer three times for 1 hr periods each, samples were post-fixed for 2 h at 25°C in darkness with 1% osmium tetroxide prepared in cacodylate buffer. The three replicates of each sample at each time point were dehydrated through an ethanol series with sequential concentrations of 70%, 95% and 100%, respectively, for 20 min each followed by infiltration and embedding in Spurr’s low-viscosity epoxy resin. Ultra-thin sections (50∼70 nm) were obtained using a Reichertultracut E microtome with a diamond knife. Sections were contrasted with 2% uranyl acetate for 30 min, followed by lead citrate staining for 30 min in total darkness before direct examination in a transmission electron microscope (Hitachi H-7500, Japan). Figures were made using an imaging system (Gatan 832CCD, USA).

### RNA extraction and cDNA-AFLP analysis

Roots of infected and un-infected plants of both susceptible and resistant genotypes were collected at 48 and 96 h post-inoculation and frozen in liquid N_2_ for storage at -80°C until use for RNA extraction and cDNA synthesis. Total RNA was extracted from 500 mg of the frozen bean root using TRNzol-A^+^ Reagent (TianGen, Beijing, China) according to the manufacturer’s instructions. The total RNA samples were treated using DNaseI (TaKaRa, Dalian, Liaoning) to eliminate residual genomic DNA. An aliquot 20 μg of extracted total RNA was used for first strand synthesis of cDNA, followed by second strand synthesis and formation of cDNA fragments using the Universal RiboClone cDNA Synthesis System (Promega, Madison, WI, USA) using manufacturer’s instruction. A total of 500 ng of double-stranded cDNA was used for template preparation as described by Bachem et al. [16].

For the AFLP reaction, the cDNAs were digested with the restriction enzymes *Eco*RI (20 units/μl) and *Mse*I (10 units/μl) (New England Biolabs, USA). The digested products of cDNA were ligated to *Eco*RI and *Mse*I adaptors: where paired *Eco*RI adaptor sequences were 5′-CTCGTAGACTGCGTACC-3′ and 3′-CTGACGCATGGTTAA-5′; and paired *Mse*I adaptor sequences were 5′-GACGATGAGTCCTGAG-3′ and 3′-TACTCAGGACTCAT- 5′. The ligated products were then pre-amplified with *Eco*RI and *Mse*I pre-amplification primers: with sequences 5′-GACTGCGTACCAATTC-3′ and 5′-GATGAGTCCTGAGTAA-3′, respectively.

Thermocycling polymerase chain reaction (PCR) involved initial denaturation at 94°C for 5 min, 25 cycles of 94°C for 30 s, 56°C for 30 s, and 72°C for 1 min for denaturing, annealing, and extension, respectively, followed by 72°C for 10 min on a T-gradient PCR Machine (Whatman Biometra, Germany). Equal amounts of pre-amplified products were diluted 1:20 in ddH_2_O, and then used as selective amplification templates. The primers used for selective amplification had various nucleotide combinations at their 3′ ends, including: *Eco*RI E-AA, E-AT, E-AG, E-AC, E-TA, E-TT, E-TG, E-TC, E-GA, E-GT, E-GG, E-GC, E-CA, E-CT, E-CG, E-CC, E-ACT and E-TGC; and *Mse*I M-AT, M-AC, M-TA, M-TT, M-TG, M-TC, M-GT, M-GG, M-CA, M-CT, M-CG, M-CC, M-GAA, M-GAT, M-GAC, M-GAG, M-GTA, M-GTC, M-GTG, M-GGA, M-GGT, M-GGC, M-GGG, M-GCA, M-GCT, M-GCC, M-GCG, M-CAA, M-CAT, M-CAC, M-CAG, M-CTT, M-CTC, M-CTG, M-CGA, M-CGT, M-CGC, M-CGG, M-CCA, M-CCT and M-CCC.

These primer combinations generated a total of 133 different combinations. Amplification of pre-amplified cDNA followed cycling conditions of initital denaturation at 94°C for 2 min then 12 cycles of touchdown PCR of 94°C for 30 s, 65°C for 30 s, and 72°C for 1 min, with a decrease in annealing temperature of 0.7°C per cycle and an additional 23 cycles of 94°C for 30 s, 56°C for 30 s, and 72°C for 1 min, followed by 72°C for 10 min. Samples were separated on 8% ureapolyacrylamide gel electrophoresis (PAGE) according to standard protocol[24] using 1×TBE buffer. The gels were directly dried at room temperature (RT) and the differential fragments were compared and marked after silver staining of the amplified DNA fragments[25] for the isolation, re-amplification and sequencing of TDF bands as described below.

### Isolation, re-amplification and sequencing of transcript-derived fragments (TDFs)

After proper comparisons of band size and staining strength, the TDFs were cut from the gel with a sterile scalpel. The cut bands were eluted using the protocol of Frost et al.[26]. The DNA fragments were immersed and stored in sterile, ultrapure water overnight, then heated to 90°C for 30 min, and isolated from gel fragments by centrifugation at RT. The eluted DNA sample (5 μl) was used with the corresponding primer combination and the same PCR reaction amplification program and volume for re-amplification. The PCR products were detected in 1.0% TAE agarose gels and visualized with a UV light. These DNA amplicons were then further purified using a TIANgel Midi Purification Kit (TianGen, China) and dissolved in 50 μl of sterile, ultrapure water for subsequent sequencing.

The TDFs purified from the agarose gels were ligated into the pMD18-T vector (TaKaRa, Japan) following the manufacturer’s instructions. The positive clones identified correctly were sequenced using M13F/R promoter primers by Sangon Biotech (Shanghai) Co., Ltd. The sequences of the TDFs were analyzed for similarity in the non redundant GenBank nucleotide database at the National Center for Biotechnology Information (NCBI) and the common reference genome database at Phytozome website (http://phytozome.jgi.doe.gov/pz/portal.html using BLAST software Blastn and Blastx (http://blast.ncbi.nlm.nih.gov/Blast.cgi). The sequences were characterized according to their homology with known nucleotide and protein sequences. The TDFs were named CBFi (Common Bean Fusarium induced) clones and were characterized for their sequence length, homology and E-value with highest hit BLAST results and genomic location by chromosome number (S1 Table) according to searches conducted in the full common bean genome database at Phytozome (http://phytozome.jgi.doe.gov/pz/portal.html).

### Analysis of TDF genes by qRT-PCR

Quantitative reverse transcription (qRT)-PCR was performed next using the 19 most promising TDFs from the sequences analysis described above. Comparisons of gene expression in root tissue samples were made based on a time series after common bean infection with *Fop* in two genotypes: BRB130 (susceptible) and CAAS260205 (resistant) collected at 24, 48, 72, 96 and 120 h post-inoculation. For each of these time-points the equivalent control, un-inoculated plant / tissue was also sampled for total RNA extract and qRT-PCR analysis. First-strand cDNA was synthesized from 1 μg of total RNA using Oligo(dT)_15_ primers supplied with the Reverse Transcription System (Promega, Madison, WI, USA) following manufacturer’s instructions.

Primers used for qRT-PCR were designed using Primer 3 online software (http://simgene.com/Primer3) based on the TDF sequences. Primer Premier 5.0 software was used to assess the optimization of the primers. Finally, qRT-PCR reactions were conducted as described by the manufacturer for the RT system. Reaction mixtures (20 μl) contained the cDNA reverse transcription solution (2μl), SuperReal PreMix (SYBR Green, TianGen, China) and 0.2μM each of each primer.

The qRT-PCR thermocycling program was 50°C for 2 min, 95°C for 15 min, 40 cycles of 10s at 95°C and 31s at 60°C. Then the melting curve temperature profile was obtained by heating to 95°C for 15s, cooling to 60°C for 30s, and slowly heating to 95°C for 15s with 0.5°C change of every 10s with continuous measurement of fluorescence at 520 nm. An *actin* gene was used as an internal control to standardize the data, and the amount of target gene transcript was normalized compared to the constitutive abundance of common bean actin[27]. All reactions were performed in triplicate, including three non-template reaction as negative controls. Quantification of gene expression was performed using a 7900 Real-Time PCR System (Applied Biosystems, Foster City, CA, USA). Threshold values (C_T_) generated from the ABI PRISM 7900 Software Tool (Applied Biosystems, Foster City, CA, USA) were employed to quantify relative gene expression using the comparative 2^-ΔΔCT^ method[28].

## Results

### Comparative analysis of disease resistance between genotypes

**Fig 1:**
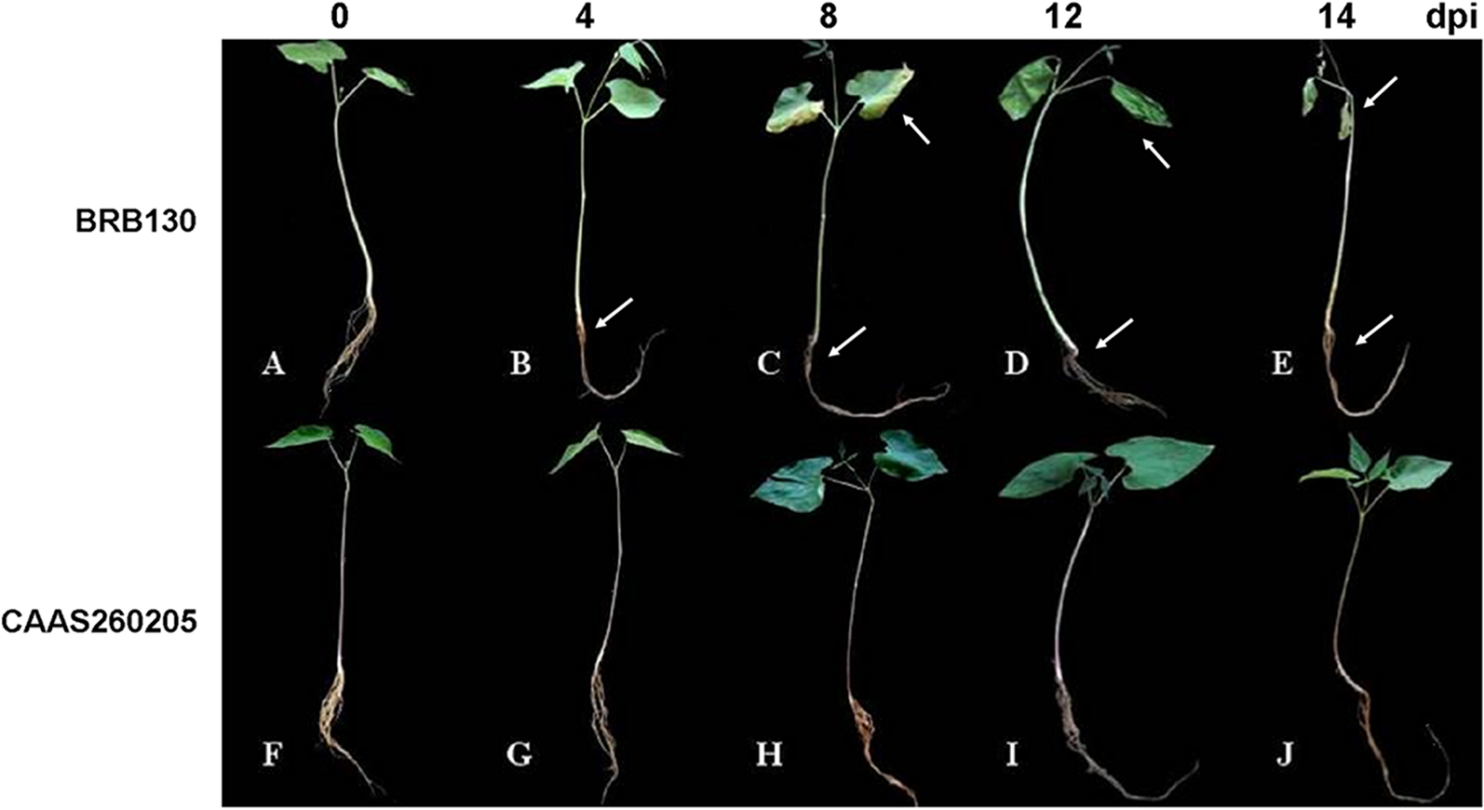
Disease severity of common bean genotypes BRB130 (susceptible, S) and CAAS260205 (resistant, R) to Fusarium wilt (*F*. *oxysporum* f. sp. *phaseoli*). A-E show the BRB130 phenotype at 0, 4, 8, 12 and 16 d post inoculation; F-J show the CAAS260205 phenotype at 0, 4, 8, 12 and 16 d post inoculation (dpi). White arrows indicate symptoms of the susceptible reaction including (i) root reddening; (ii) hypocotyl browning; (iii) leaf chlorosis; (iv) stem and leaf wilting.

Our first notable results were the phenotypic differences between *Fop* susceptible and resistant genotypes. Infected roots of susceptible BRB130 plants showed the typical reddening color of Fusarium wilt even at 4 days postinoculation (dpi) compared with the control (non-inoculated) genotypes. Weak roots, leaf chlorosis, stem browning and slight drooping of cotyledons were other symptoms visible in seedling stage. The next wilt symptoms appeared on leaves and the main stem at 8 dpi in the infected, wilt-susceptible BRB130 plants (Fig 1). The resistant genotype CAAS260205, in contrast, showed normal growth in comparison to BRB130 and other non-resistant genotypes (data not shown) as well as the uninoculated plants even at 16 dpi. CAAS260205 did show a slight stunting and reddening of the roots (Fig 1), indicating that resistance was at the level of preventing fungal colonization of the remaining parts of the plant.

**Fig 2:**
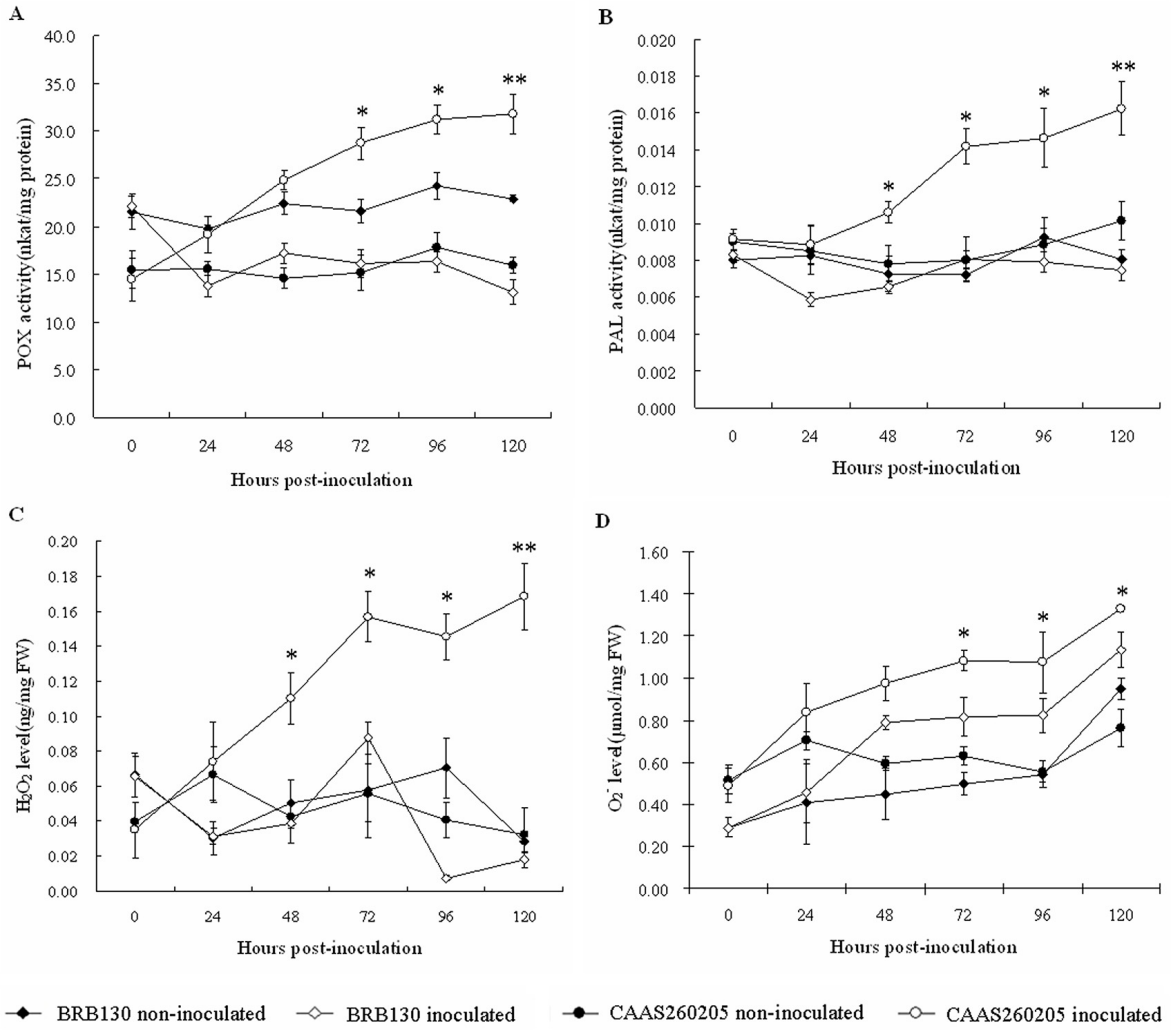
Defense responses in the roots of susceptible genotype BRB130 and resistant genotype CAAS260205 common bean seedlings inoculated with *Fusarium oxysporum* f. sp. *phaseoli* versus roots of non-inoculated (control) seedlings at 0, 24, 48, 72, 96 and 120 h post-inoculation. A: POX activities; B: PAL activities; C: H_2_O_2_ accumulation; D: O_2_^-^ accumulation. Results from three repeated experiments are displayed. Bars indicated the standard deviations (± SD). Probability values between the control and treated plants were estimated by the Student’s *t* test, with significant difference at *P0.05 and **P0.01.

In order to determine the biochemical and physiological difference between the defense responses to *Fop* infection of susceptible and resistant common beans, the activity of the enzymes PAL and POX were assayed in the roots (Fig 2A, Fig 2B). PAL and POX activity of infected CAAS260205 and BRB130 roots increased after 24 h in comparison with a reduction in non-inoculated plants. PAL and POX activity both showed large genotype differences starting at 48 h to 120 h after inoculation, reaching a maximum for both enzymes in infected CAAS260205 compared to infected BRB130 at those times. On the other hand, the levels of H_2_O_2_ and O_2_^-^, the indicators of reactive oxygen species, were significantly higher in infected CAAS260205 than in infected BRB130 (Fig 2C, Fig 2D). Both H_2_O_2_ and O_2_^-^ levels peaked in infected CAAS260205 at 120 h and were higher than in infected BRB130. However, the H_2_O_2_ and O_2_^-^ concentrations were not significantly different than the uninfected controls for each genotype, respectively.

**Fig 3:**
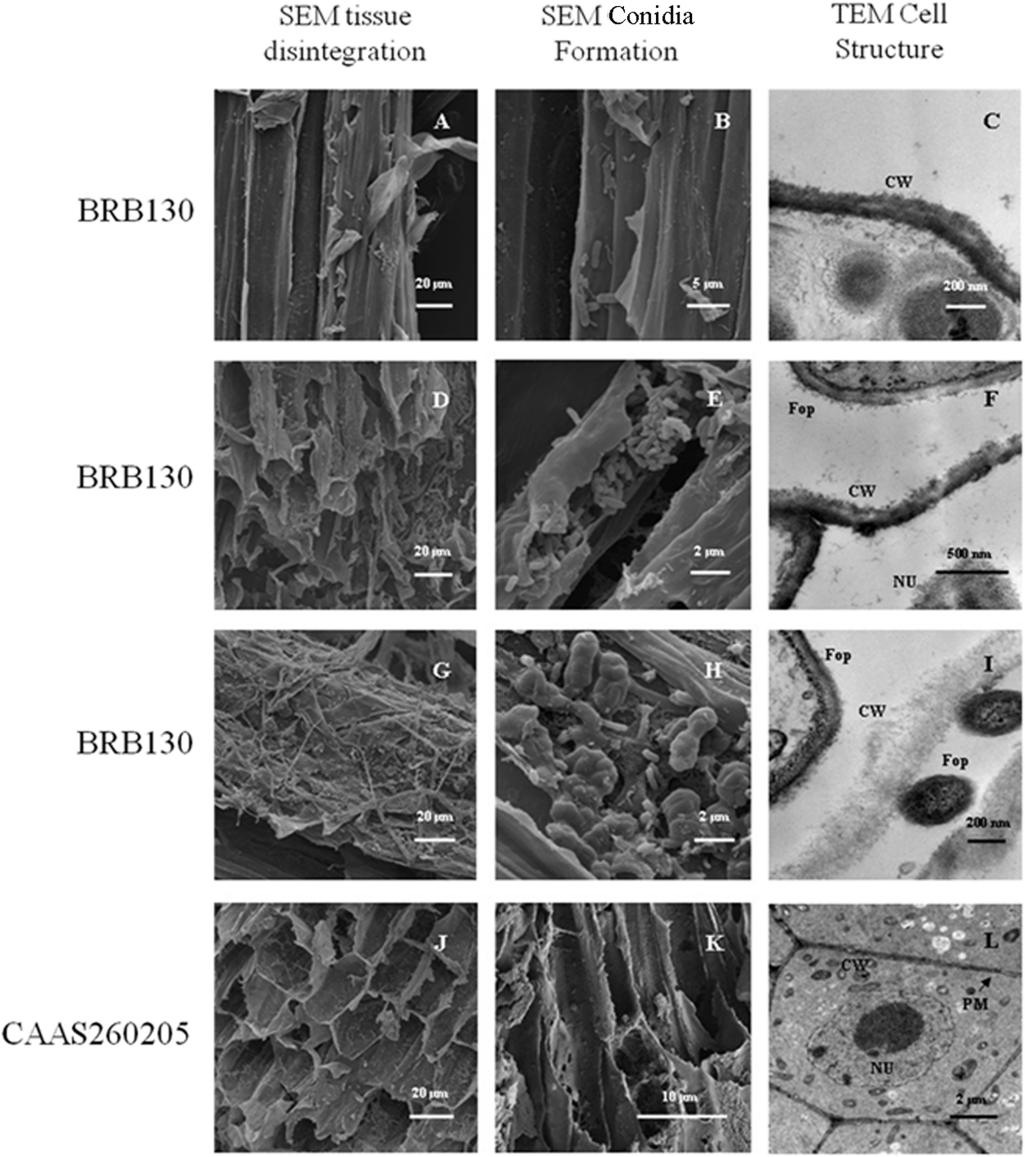
Electron micrographs of infected root tissues of Fusarium wilt infected resistant and susceptible common bean plants. Scanning electron microscopy (SEM) of root sections from infected BRB130 plants at 4, 8, 12 days after infection showing tissue disintegration (A, D, G) and conidia formation (B, E, H). SEM of root sections from infected CAAS260205 plants at 12 days after infection showing infected xylem vessels (J), but tissue damage and conidia formation at 21 days after infection (K). In addition, transmission electron microscopy (TEM) of root sections from infected BRB130 plants at 4, 8, 12 days after infection showing cell wall disintegration (C, F, I). Infected CAAS260205 plants at 21 days after infection showing a single integral xylem cell (L); Cell wall = CW; Plasmodesma = PM; Nucleus = NU. The pathogen hyphae of *F*. *oxysporum* f. sp. *phaseoli* isolate FOP-DM01 are indicated as *Fop* in the sub-figures (F) and (I).

Scanning electron microscopy (SEM) further confirmed the results of fungal infection in the infected susceptible genotype and the time of onset of tissue damage (Fig 3A). For example, fungal microspores were visible inside the xylem of infected BRB130 plants at 4 d post-inoculation (Fig 3B). Further SEM microscopy evaluations of root sample cross-sections showed that all vascular tissues were colonized after 4 d of inoculation with *Fop* in the infected treatments (Fig 3C). The vascular tissue damage was even more evident at 8 d (Fig 3D), and a larger number of spores were found in the xylem (Fig 3E). Close examination of infected root ultrastructure by transmission electron microscopy (TEM) showed that the pathogen colonization of the host was accompanied by a marked wall modification including primary wall alteration and cytoplasm dissolution (Fig 3F). At 12 d, the original tissue architecture was almost destroyed (Fig 3G) and the fungal spores increased in number (Fig 3H). While the plant host tissues underwent a complete degradation, the pathogen hyphae did not exhibit any apparent disorganization (Fig 3I). The observed disruption of root cells coincided with the occurrence of macroscopically visible symptoms leading to plant death. Meanwhile, infected root sections of CAAS260205 showed no obvious damage even after 12 d (Fig 3J). Furthermore, fungal spores were not detected in the xylem vessels of this genotype, although there was some amount of fungal colonization with slight tissue disintegration at 21 d (Fig 3K). The root cortical parenchyma cells exhibited few structural modifications in the resistant genotype compared to the susceptible one, and the integrated structure of cell wall and nucleus were still observed (Fig 3L).

### Screening of defense-related genes in common bean by cDNA-AFLP

Differential transcript profiles of the cDNA-AFLP analysis were carried out between infected and non-infected tissues of both BRB130 and CAAS260205 (S1 Fig). Approximately 8,730 fragments ranging from 25 to 400 base pairs (bp) were generated from 768 primer combinations (2N×2N and 3N×2N) and genomic representations produced with two restriction sites: *EcoR*I and *Mse*I. Differentially expressed fragments which showed differential intensity after pathogen infection of susceptible and resistant samples compared to their non-inoculated controls were marked and selected for further analysis. A total of 423 highly reproducible bands (40–400 bp) were either uniquely present in infected BRB130 or CAAS260205 plants or amplified to a greater extent in these genotypes when comparing infected with non-inoculated plants. The distinctly up- and down-regulated bands were excised, cloned and sequenced from both ends to obtain full amplicon sequences. In the next step, 122 of these 423 bands were re-amplified with specific primer combinations and eluted from gels to check the fragment sizes. Meanwhile, the sequences were deposited into GenBank at NCBI in two groups with accession numbers starting at JZ468984 and JZ822416, respectively (S1 Table).

### Functional classification of transcript-derived fragments (TDFs)

Following the careful selection of meaningful TDFs based on the comparison of infected and control tissues 122 cDNA-AFLP fragments were cloned and sequenced and analyzed for homology to know genes. Of these, 98 (80% of the total TDFs) showed moderate to high sequence homology (75 to 100%) with known or predicted genes deposited in the NCBI GenBank nucleotide database and the common reference genome database at Phytozome website (S1 Table). Meanwhile 24 other sequenced TDFs were not similar to known genes of which 10 (8.2%) were considered homologous with expressed proteins with unknown functions from other organisms. The remaining 14 (11.5%) TDF fragments showed no or very little homology with known genes found in any other organisms. Of the genes with homologous sequences, 98 TDFs showed different expression patterns between resistant and susceptible tissues, 8 TDFs showed reduced or non-enhanced expression upon infection by *Fop* and 13 TDFs showed enhanced expression in both genotypes when infected. In 58 out of the 98 TDFs, the expression levels were induced in CAAS260205 roots but not in BRB130 roots. Chromosomal location of all 122 TDFs were found in the Phytozome common bean genome database.

**Fig 4:**
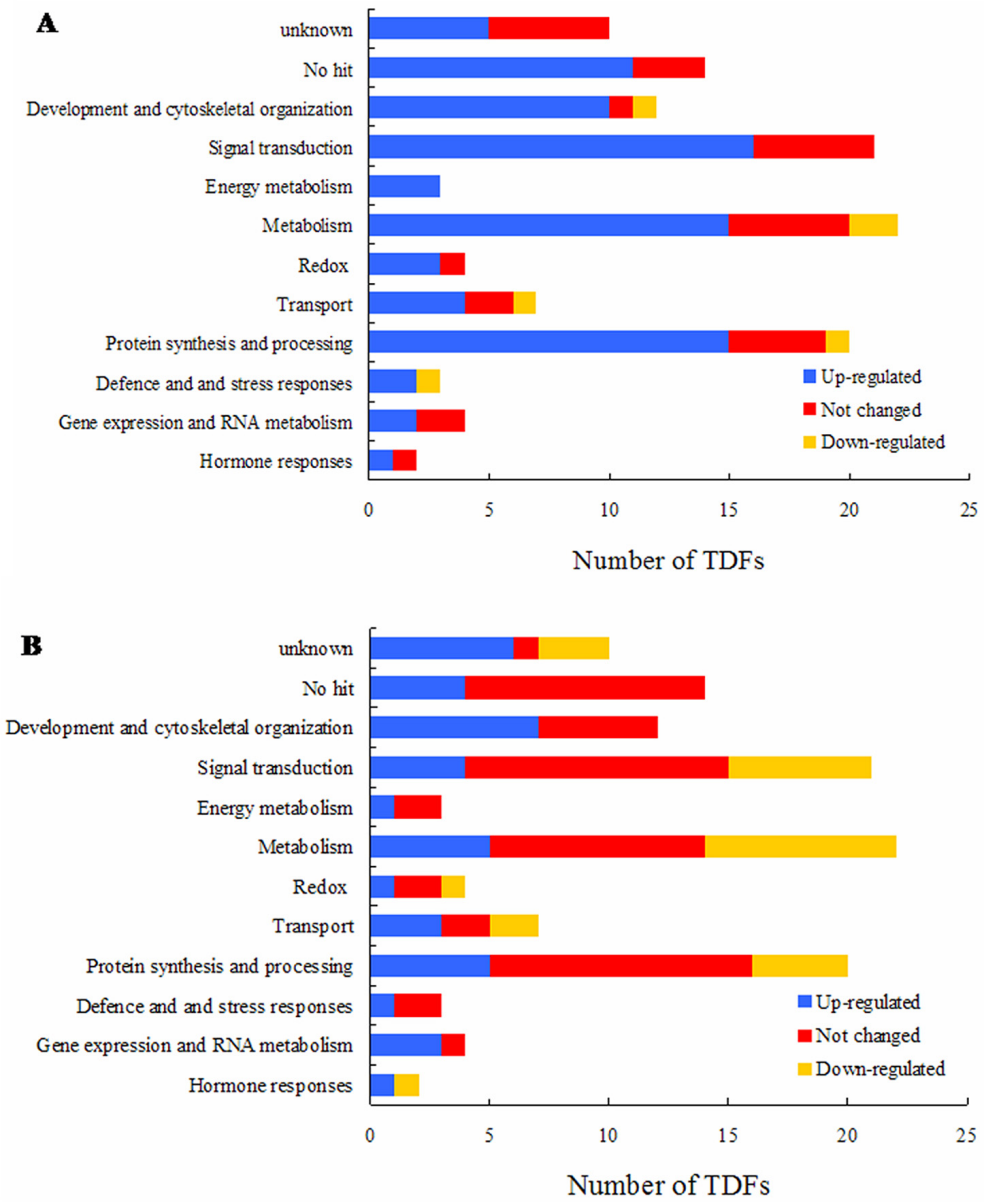
Comparison of transcript-derived fragments (TDFs) homology classification for Fusarium wilt resistant and susceptible common bean genotypes across functional categories. (A) Resistant genotype CAAS260205 and (B) Susceptible genotype BRB130.

Among the TDFs with homology to known genes, 2 fragments were parts of hormone response genes, 4 were part of redox-related genes, 7 were part of transport-related genes, 4 were part of gene expression and RNA metabolism-related genes, 3 were part of defense and stress response-related genes, 20 were part of protein synthesis or processing-related genes, 25 were part of general or energy metabolism genes, 21 were part of signal transduction genes and 12 were part of genes related to development and cytoskeletal organization (S2 Fig). Compared to the susceptible genotype BRB130, the resistant genotype CAAS260205 had more up-regulated root TDFs across the primary functional categories found in the similarity search (Fig 4), while 19 genes showed no change or suppressed expression in CAAS260205.

### Experimental verification of TDFs by qRT-PCR

**Fig 5:**
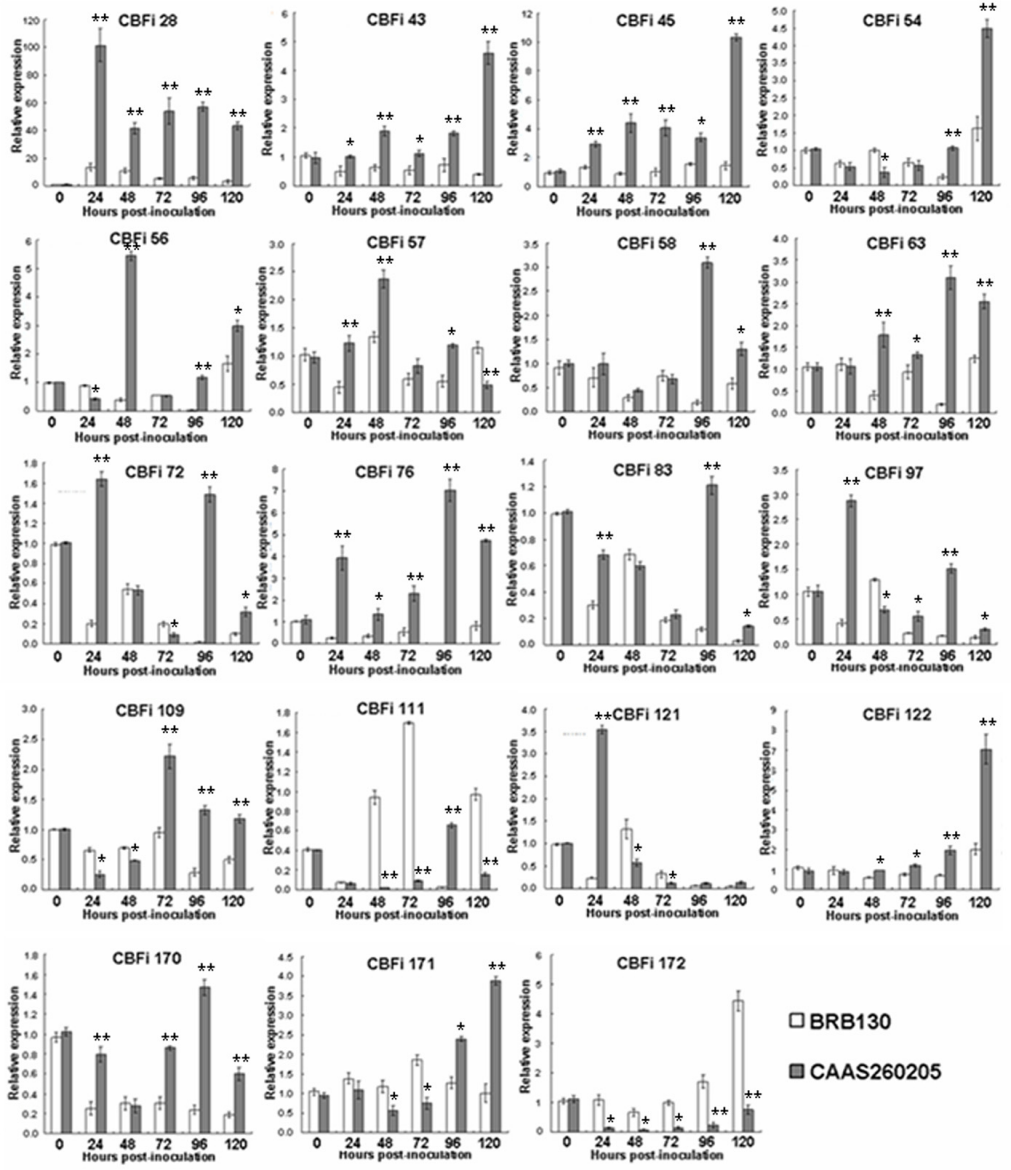
Quantitative real-time PCR (qRT-PCR) analyses of 19 selected transcript-derived fragments (TDFs) in response to *F*. *oxysporum* f. sp. *phaseoli* infection in susceptible BRB130 and resistant CAAS260205 common bean genotypes. Probability values between infected BRB130 and CAAS260205 plants were estimated by the Student’s *t* test, with significant difference at *P0.05 and **P0.01.

To assess the reliability of the cDNA-AFLP technique to identify disease response genes from the *Fop*-common bean pathosystem, we monitored the expression of candidate TDFs during the development of Fusarium wilt on the plant host. The relative expression levels of 19 genes selected from the early stages of plant pathogen interaction were determined by qRT-PCR reactions (Fig 5). These were performed not only to validate the results from the cDNA-AFLP analysis but also to quantitatively assess the relative abundance of the transcripts at different time points (0, 24, 48, 72, 96 and 120 h) in the infected roots of both BRB130 and CAAS260205. The TDFs of interest were selected on the basis of their intensity and differential pattern of expression in the cDNA-AFLP experiment. Sequences of TDFs with significant homology to the known gene and protein sequences from the GenBank database were used to design the specific quantitative analysis primers (S2 Table). All of these 19 genes showed an expression pattern roughly consistent with the prediction based on the cDNA-AFLP analysis. As shown in Fig 5, the transcripts of the candidate CBFi genes responding to different time points were detected in resistant CAAS260205 and susceptible BRB130 but at different levels. CBFi28 (similar to GH3 auxin-regulated protein), CBFi43 (similar to ubiquitin-like protein), CBFi45 (similar to polyubiquitin) and CBFi76 (similar to secretory peroxidase) showed enhanced expression in inoculated CAAS260205 compared with inoculated BRB130 at every time point after inoculation. In contrast, CBFi172 (similar to 14-3-3 protein) showed consistent and low expression in challenged CAAS260205 until 120 h after infection, whereas infected BRB130 showed enhanced expression after 96 h post-infection. Likewise, CBFi56, CBFi63 and CBFi122 were found to be up-regulated in inoculated CAAS260205 after 48 h of pathogenic treatment compared with BRB130, where they were down-regulated or constant.

Meanwhile, the expression of four other genes, CBFi54, CBFi58, CBFi83 and CBFi171 in CAAS260205 was dramatically increased after 96 h of infection, at the same time that infected BRB130 showed reduced expression. Similarly, but with an earlier peak in expression, the genes CBFi111 and CBFi170 increased in CAAS260205 only at 96 h of infection but then decreased at 120 h post-infection. In contrast, infected BRB130 showed enhanced expression for CBFi111, and low expression for CBFi170 throughout the infection period. Two more genes, CBFi57 and CBFi121, had increased expression levels only at 24 h of infection in CAAS260205, decreasing sharply thereafter until 120 h after infection. The same genes had only low expression levels in the infected BRB130 genotype. One additional pattern was observed for CBFi109 which had an increase in expression level only at 72 h for infected CAAS260205, but then decreased compared with the expression level in BRB130 after infection. Finally, CBFi72 and CBFi97 expression in CAAS260205 increased at 24 h post-infection, and then decreased significantly with the expression levels enhanced again at 96 h. In contrast, BRB130 showed low expression level for these last three genes across all the time periods of infection.

## Discussion

The disease resistant genotype used for this study was CAAS260205, which is a common bean landrace originally from Yunnan, China, characterized by high yield, high quality and high resistance to a local isolate of the Fusarium wilt pathogen. The higher activities of defense enzymes such as PAL and POX, and higher level of both H_2_O_2_ and O_2_^-^ in infected CAAS26205 tissue compared to the susceptible genotype BRB130 may explain its high level of resistance. Limiting the *Fop* infection to the roots may be one of the strategies that the resistant genotype uses to prevent death from Fusarium wilt.

The comparison of BRB130 and CAAS26205 was used to investigate the profile of gene expression in the interaction between *Fop* and common bean at different stages of the infection process, and provided additional evidence for an active resistance response in the resistant genotype. The result of expression analysis shows that the resistant genotype responded to the pathogen infection at a later time point than the susceptible genotype. Furthermore, expression of most defense-related genes analyzed was relatively lower in CAAS260205 than in BRB130 in the earlier stages of infection, but higher in later stages.

Differential display was used to discover genes important to the *Fop*-common bean pathosystem. In this part of the study, we carried out a cDNA-AFLP based comparative analysis of infected and uninfected tissues of resistant and susceptible individuals. Our data showed that the expression level of most TDFs was higher in CAAS260205 than in BRB130, and these TDFs were associated with different defense-related or growth-regulatory genes. Among the pathogen-induced differential bands, 81.9% had enhanced expression in the inoculated CAAS260205 tissue compared to the inoculated BRB130 tissue, whereas only 18.1% showed no change or suppression in the infected CAAS260205 compared with the infected BRB130. Some examples of the induced genes from CAAS 260205 are discussed below based on their functional role in disease resistance responses.

Three TDFs encoding different protein kinases (PKs) were identified. Protein kinases are known to play a central role in signaling during pathogen recognition and the subsequent activation of plant defense mechanisms[29,30]. They are sometimes found as part of classical R genes with recognition domains of the leucine rich repeat (LRR) class but are also found alone as single functional PK domain proteins[31]. In our study, CBFi109 and CBFi134 were highly homologous to serine-threonine kinase genes from *Phaseolus vulgaris* and *Lotus japonicus*, which were overexpressed in infected wilt-resistant genotypes during the interaction. CBFi155 encoded a receptor-like kinase similar to the *Glycine max* clone cw64.

The role in disease resistance response of protein kinases and receptor like kinases with PK domains combined with LRRs is well-known[31]. Several PK or PK domain containing genes were among the first resistance genes to be cloned by map-based cloning including *Pto* from *Lycopersicon esculentum* [32,33]. However most confirmed and homology predicted resistant genes have been LRR-PK or LRR genes in the TIR and non TIR categories. In a recent example Chaparro-Garcia (2011) demonstrated that *N*. *benthamiana NbSERK3* which is a leucine-rich repeat (LRR) receptor-like kinase BAK1/SERK3 significantly contributes to resistance to *P*. *infestans* and regulates the immune responses triggered by the *P*. *infestans* PAMP protein INF1[34]. Surprisingly we identified only one LRR genes in our analysis, perhaps because they are at low expression levels while PKs may be expressed at higher levels that are detectable in differential display. Lu et al. (2010) identified a receptor-like cytoplasmic kinase BIK1 that is rapidly phosphorylated upon flagellin perception, associates with a flagellin receptor complex to initiate innate plant immunity[35].

Another point of entry for disease resistance genes are those controlling calcium location in the cells[36]. The genes CBFi58 and CBFi42 had homology with calcium-binding protein and calcium-dependent protein kinase and were interesting candidate genes in this regard. A previous study revealed a prominent role for calcium and calmodulin (CaM), a calcium-binding protein, in the regulation of SA accumulation and signaling[36]. Binding of calcium/CaM to the *Arabidopsis* transcription factor SR1 suppresses plant defense.

Conversely, binding of CaM to CaM-binding protein is positively correlated with pathogen resistance[36,37]. A calcium-activated protein kinase also played a crucial role in the responses to wounding and pathogen attacking in tomato plants. The plasma membrane H^+^-ATPase in tomato was a possible target of the Ca^2+^-dependent protein kinase which then activates the defense-related signaling pathway[38].

Plant hormones, such as jasmonate (JA), auxin, abscisic acid (ABA) and salicylic acid (SA), have also been implicated in plant host resistances to pathogens[39]. In this study, we found five TDFs involved in the mechanism of plant hormone regulation, they were CBFi61, CBFi28, CBFi63, CBFi111 and CBFi155. These five genes belonged, respectively, to the JA, auxin, ABA and SA-dependent pathways and can be implicated to play a role in the plant’s defense response. In the first case, CBFi61 was found to encode an F-box protein with similarity to the *COI1* gene of Arabidopsis, which appear to regulate defense against pathogens by targeting repressor proteins for removal by ubiquitination[40].

In the second example, CBFi28 shared similarity with the *Glycine max* GH3 gene and was annotated as an auxin-regulated protein that in our study was dramatically up-regulated in infected tissues at all time points. Rice *GH3-8*, an auxin-responsive gene functions in an auxin-dependent development pathway to activate disease resistance without the need for SA and JA induced pathways. Overexpression of *GH3-8* results in enhanced disease resistance to the rice pathogen *Xanthomonas oryzae* pv *oryzae* [41].

The next two genes, CBFi63 and CBFi111, encoded a protein phosphatase type 2C and a DEAD box helicase, respectively. Protein phosphatase type 2C is a negative regulator of ABA responses which is required for plant defense[42]. ABA has been proven to increase susceptibility by counteracting SA-dependent defenses, and ABA-dependent priming of callose biosynthesis promotes enhanced resistance to some pathogens[43]. Meanwhile, DEAD-box RNA helicases have been reported to play an important role during development and stress responses in various organisms[44, 45]. Rice *OsBIRH1*, encoding a DEAD-box RNA helicase was shown to function in defense responses against pathogen infection and oxidative stresses[46].

The fifth case and final hormone related gene, CBFi155, encoded a receptor-like kinase which works as one of various protein kinases in plant SA-dependent pathways. Komjanc et al. (1999) isolated a leucine-rich repeat receptor-like PK gene which was induced both by *Venturia inaequalis* infection and SA treatment in *Malus* × *domestica*[47]. CBFi55 could also be classified in the PK group but had a LRR and homology to the SA responsive genes and therefore was classified as hormone related. Overall, our results indicated that ABA should be expected to play a negative role in the resistance response to *Fop* infection while SA and JA was expected to be a positive regulator.

Most plant disease resistance (*R*) genes known today encode proteins with a central nucleotide binding site (NBS) and a C-terminal Leu-rich repeat (LRR) domain and this is also the case in common beans[48,49,50]. Interestingly, only one *R* gene-like gene was discovered in this study with those domains although one PK-LRR gene was discovered as described above. The NBS = LRR gene CBFi171 was interesting because it was homologous with a NBS-LRR-like sequence from common bean that has been implicated in major gene (*Co-2*) resistance to the anthracnose pathogen (*Colletotrichum lindemuthianum*)[51].

The CBFi71 gene mapped in the Phytozome reference whole genome sequence for common bean to chromosome 11 which is where a possible cluster of Anthracnose resistance genes and quantitative trati loci for anthracnose resistance including *Co-2* is located. Therefore, further genetic and plant pathology research is needed to determine if this gene or any others of the NBS-LRR class are major resistance genes that are involved in resistance to specific isolates of the Fusarium wilt pathogen in common bean.

Besides the most abundant groups mentioned above, there were additional TDFs that may reflect the action of other defense systems in resisting the attack of the *Fop* pathogen. These genes included several encoding a ubiquitin-like protein (CBFi43), a poly-ubiquitin protein (CBFi45) and a ubiquitin-protein ligase (CBFi170). One study demonstrated that protein degradation mediated by ubiquitination played an essential role not only in JA but also in auxin signaling[52].

The JA and auxin signaling pathways probably work together in the activation of defense responses to pathogen attack and resistance to other stresses in plants[11,23,52].

Additionally, several TDFs from this study would be novel in terms of their mechanism of Fusarium wilt control if they do play such a role in disease resistance. These genes included one for a 14-3-3 membrane protein (CBFi172), one for a secretory peroxidase (CBFi76) and another for a non-symbiotic hemoglobin (CBFi9). Some of the novel proteins have been confirmed to be associated with plant disease resistance in other pathosystems but not in Fusarium wilt.

The 14-3-3 proteins have a potential role in acting as receptors of fungal toxins[53]. The up-regulation of CBFi72 and other 14-3-3-like molecules strengthens the evidence for their role in attaching to fungal toxins and subsequently causing irreversible opening of stomata leading to wilt of the susceptible genotype[54].

Meanwhile, peroxidases are known to play an important role in plant defense response to the attack of a host tissue by many pathogens. Based on the sequence of CBFi76, a secretory root-expressed peroxidase gene has been isolated from common bean infected with *Fop* and was suggested to provide a diagnostic marker for selection of resistance to this disease[55].

The CBFi19 gene with similarity to a cotton non-symbiotic hemoglobin GhHb1 protein could be suggested to play a role in the defense responses against pathogen invasions, possibly by modulating the NO level and the ratio of H_2_O_2_/NO in the defense reponse process[56].

In summary, this study has provided a valuable new insight into the molecular mechanisms of resistance to Fusarium wilt in common bean by reporting 122 newly-identified differential expression fragments in common bean seedling roots most of which were differentially expressed in response to *Fusarium oxysporum* f. sp. *phaseoli*. The qRT/PCR results validated the cDNA-AFLP reactions where polymorphic fragments represented genes that were up-regulated and these were for the same time points of 48 and 96 h after infection. Comparing these time points with uninfected root tissue, the expression of all TDFs we selected were induced at either of the timepoints and most were increased at both stages.

Most importantly, this is the first report of expression of a large number of gene fragments related to the defense response in common bean challenged by *F*. *oxysporum* f. sp. *phaseoli*. The 122 TDFs were found to be encoded in different regions across the genome with especially good representation on chromosome 2 (20), chromosome 7 (18) which have been marker rich in past studies[57–59]. Meanwhile, intermediate numbers of TDFs were found on chromosome 3 (10) chromosome 6 (10), chromosomes 1 and 8 (12 markers each) and chromosome 9 (13). Fewer TDFs were on chromosome 4 (5), chromosome 5 and 10 (7 each) and chromosome 11 (8) which distinguish these markers from NBS-LRR based markers which are biased to chromosomes 2, 4 and 11[49,60], the last of these linkage groups containing the Co-2 gene and many NBS-LRR homologs [61].

In a final conclusion, the good genomic distribution of the TDFs would make them very useful as molecular markers for tagging disease resistance genes in beans or correlating with QTL for Fusarium wilt resistance. Therefore, the TDFs described here should be very useful for the development of genetic markers for screening, identification and breeding of new disease-resistant genotypes and constitute a solid foundation for future research in this pathosystem.

## Supporting Information

S1 Fig Selected cDNA-AFLP profiles of non-inoculated and inoculated BRB130 and CAAS260205 common bean root samples using different selective primer pair combinations with arrows indicating some of the bands selected for further analysis. (TIF)

S2 Fig Classification into functional groups for clone sequences from transcript-derived fragments (TDFs) based on Fusarium wilt-infected or non-infected common bean root tissues used for cDNA-AFLP reactions from the susceptible and resistant genotypes, BRB130 and CAAS260205, respectively using BLASTX and BLASTN. Percentages represent proportion of total TDFs found in each of 12 categories. Numbers in parentheses denote the actual number TDFs identified within each category.
(TIF)

S1 Table Analysis of transcript-derived fragments found by cDNA-AFLP of *Fusarium oxysporum* f. sp. *phaseoli*-infected common bean root tissue. (DOC)

S2 Table Primers used for 19 target cDNA clones for real time quantitative polymerase chain reaction analysis (qRT-PCR) in *F*. *oxysporum* f. sp. *phaseoli* infected susceptible BRB130 and resistant CAAS260205 common bean genotypes. (DOC)
