# Supplementary figures and images for: Differentially Expressed Genes in Resistant and Susceptible Common Bean (Phaseolus vulgaris L.) Genotypes in Response to Fusarium oxysporum f. sp. phaseoli
Source: PLoS One. 2015 Jun 1;10(6):e0127698. doi: 10.1371/journal.pone.0127698 (PMC4452237; doi:10.1371/journal.pone.0127698)

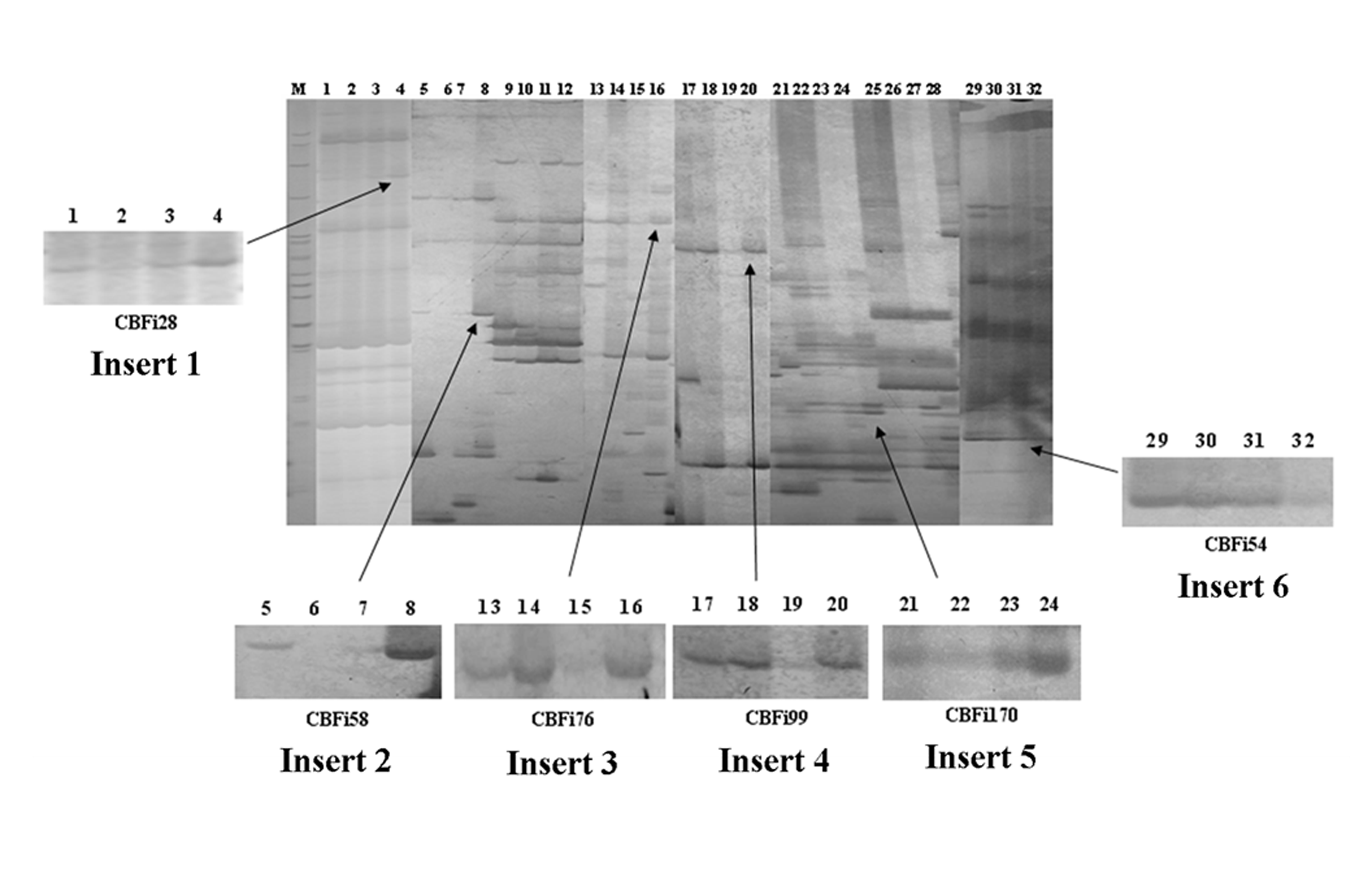

Supplement: S1 Fig — (TIF) [file pone.0127698.s001.tif]

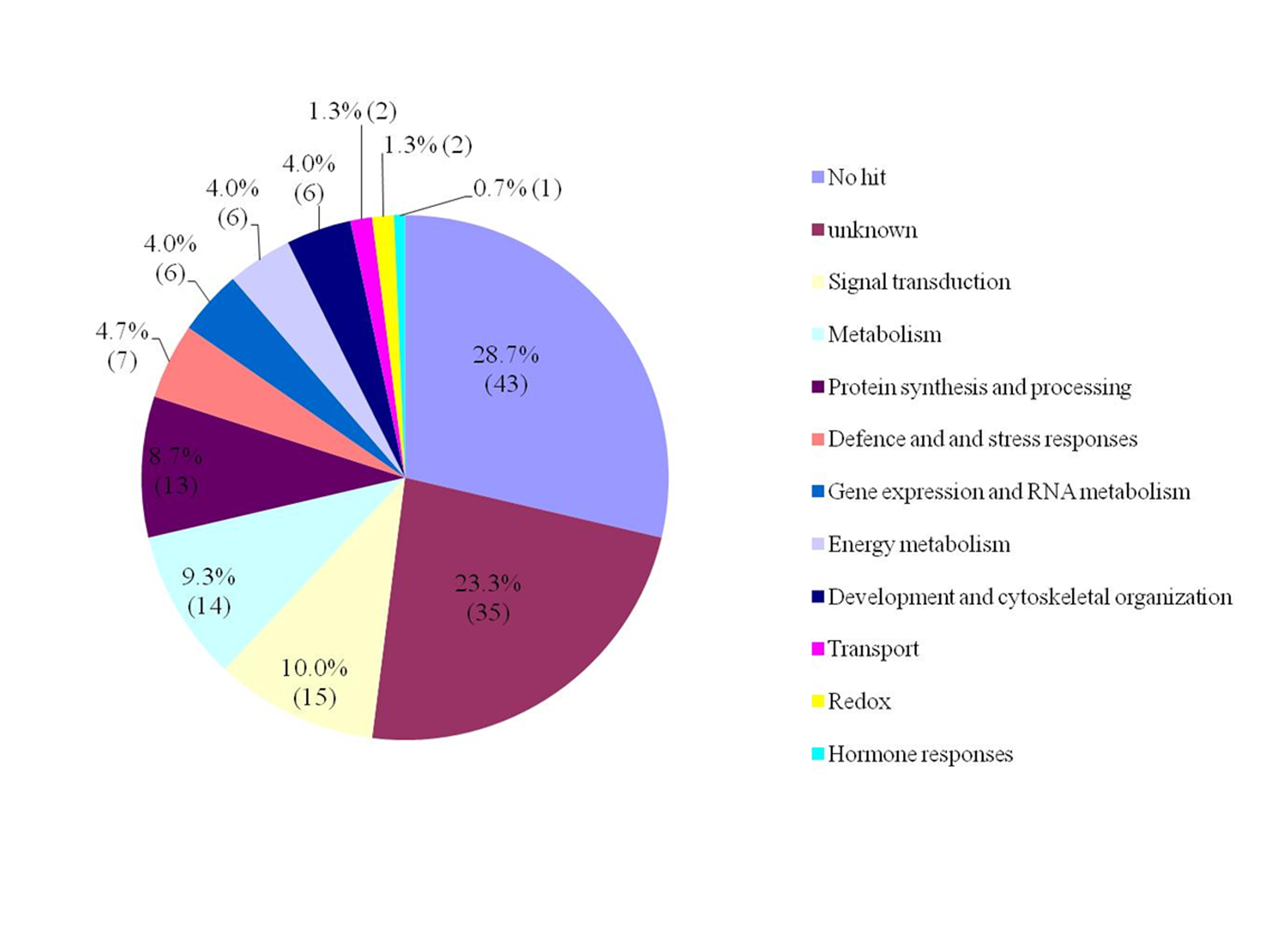

Supplement: S2 Fig — Percentages represent proportion of total TDFs found in each of 12 categories. Numbers in parentheses denote the actual number TDFs identified within each category. (TIF) [file pone.0127698.s002.tif]
