# Supplementary material for: Differentially Expressed Genes in Resistant and Susceptible Common Bean (Phaseolus vulgaris L.) Genotypes in Response to Fusarium oxysporum f. sp. phaseoli
Source: PLoS One. 2015 Jun 1;10(6):e0127698. doi: 10.1371/journal.pone.0127698 (PMC4452237; doi:10.1371/journal.pone.0127698)
Supplement: S1 Table — (DOC) [file pone.0127698.s003.doc]

**Table S1**. Analysis of transcript-derived fragments found by cDNA-AFLP of *Fusarium oxysporum* f. sp. *phaseoli*-infected common bean root tissue

| Clone ID | Length  (bp) | GB^a^ Accession | Primer combination | level of expression^b^ | | Homology^c^ | Genome location^d^ | Identity (%) | E-value^e^ |
| --- | --- | --- | --- | --- | --- | --- | --- | --- | --- |
|  |  |  |  | CAAS  260205 | BRB130 |  |  |  |  |
| **Metabolism** | | | | | | | | | |
| CBFi4 | 193 | JZ468984 | E-AT/M-GT | +++ | + | *Trifolium repens* voucher NC A88 beta-glucosidase-like protein gene, partial cds (EF990485) | 11 | 82 | 1e-29 |
| CBFi5 | 125 |  | E-AT/M-GT | + | NC | Phospholipase D (Phvul.002G104200.1) | 02 | 100 | 2.4e-40 |
| CBFi22 | 120 | JZ468986 | E-TG/M-CA | NC | + | *Glycine max* glyceraldehyde-3-phosphate dehydrogenase B subunit mRNA, complete cds (DQ224371) | 09 | 98 | 8e-17 |
| CBFi25 | 144 | JZ469058 | E-CA/M-CG | NC | + | UDP-glucuronosyl and UDP-glucosyl transferase (Phvul.008G034000.1) | 08 | 100 | 2.6e-53 |
| CBFi36 | 84 |  | E-TA/M-GGA | NC | ++ | Glutamine synthetase (Phvul.007G152500.1) | 07 | 100 | 4e-22 |
| CBFi38 | 344 | JZ468990 | E-TC/M-GAC | － | NC | *Glycine max* (clone pSAT2) nuclear-encoded mitochondrial aspartate aminotransferase mRNA, complete cds (L40579) | 08 | 95 | 3e-55 |
| CBFi49 | 366 | JZ469060 | E-CC/M-GGT | NC | － | Acetyltransferase (GNAT) family (Phvul.002G044100.1) | 02 | 100 | 1.5e-55 |
| CBFi50 | 137 |  | E-TT/M-GCA | NC | － | Phospholipase D (Phvul.005G177300.1) | 05 | 100 | 2.4e-42 |
| CBFi53 | 366 | JZ468996 | E-GC/M-CAA | ++ | NC | *Cicer arietinum* mRNA for cytosolic malate dehydrogenase (ORF1), clone CanCMDH (AJ299059) | 07 | 91 | 1e-53 |
| CBFi54 | 172 | JZ468997 | E-CT/M-GCT | － | NC | 3-hydroxyacyl-CoA dehydrogenase (Phvul.003G096500.1) | 03 | 100 | 3.7e-65 |
| CBFi57 | 144 | JZ468999 | E-AT/M-GCG | ++ | NC | *Glycine max* alanine aminotransferase 3 (AlaAT3) mRNA, complete cds (EU165373) | 02 | 90 | 6e-26 |
| CBFi64 | 107 |  | E-AT/M-GCC | +++ | NC | Sterol regulatory element-binding protein (Phvul.007G206000.1) | 07 | 100 | 9.3e-32 |
| CBFi70 | 183 |  | E-CG/M-GCT | + | NC | Shikimate dehydrogenase (Phvul.006G096400.1) | 06 | 100 | 2.7e-67 |
| CBFi72 | 169 | JZ469004 | E-CG/M-CAT | ++ | － | *Glycine max* phosphomannomutase mRNA, complete cds (DQ442994) | 08 | 94 | 1e-54 |
| CBFi79 | 189 | JZ469008 | E-CG/M-CAT | + | － | AMP deaminase (Phvul.010G078200.1) | 10 | 100 | 1e-15 |
| CBFi80 | 136 | JZ469067 | E-AG/M-CGC | ++ | NC | N-terminal glutamine amidase (Phvul.007G252600.1) | 07 | 100 | 1.3e-50 |
| CBFi114 | 63 |  | E-TT/M-GCC | ++ | － | Methyltransferase (Phvul.010G105300.1) | 10 | 100 | 2.2e-10 |
| CBFi126 | 64 | JZ469027 | E-CA/M-GAG | + | － | Metalloprotease M41 ftsh (Phvul.007G116400.1) | 07 | 100 | 5.3e-12 |
| CBFi142 | 96 | JZ469036 | E-TC/M-GCT | ++ | + | *Ricinus communis* Adaptin ear-binding coat-associated protein, putative, mRNA (XM002525073) | 05 | 82 | 3e-08 |
| CBFi149 | 115 | JZ469041 | E-CA/M-GAC | + | － | *Glycine max* mRNA for peroxisomal 3-ketoacyl-CoA thiolase precursor, complete cds (AB333797) | 07 | 91 | 4e-26 |
| CBFi153 | 129 | JZ469042 | E-TC/M-GCT | ++ | NC | *Medicago sativa* trehalose-6-phosphate phosphatase mRNA, complete cds (HM156211) | 09 | 84 | 9e-23 |
| CBFi169 | 170 | JZ469052 | E-AT/M-GCC | + | － | *Ricinus communis* 6-phosphogluconolactonase, putative, mRNA (XM002512016) | 03 | 85 | 2e-32 |
| **Protein synthesis and processing** | | | | | | | | | |
| CBFi19 | 128 |  | E-CC/M-TA | NC | + | ATP-dependent RNA helicase (Phvul.010G097700.1) | 10 | 100 | 5.7e-42 |
| CBFi24 | 247 | JZ469057 | E-TT/M-CC | + | NC | 40S ribosomal protein S25 (Phvul.009G022600.1) | 09 | 100 | 1.6e-65 |
| CBFi32 | 174 | JZ469059 | E-AA/M-GTG | NC | +++ | O-sialoglycoprotein endopeptidase (Phvul.006G109300.1) | 06 | 100 | 4e-71 |
| CBFi43 | 347 | JZ468992 | E-CC/M-GAC | + | － | *Phaseolus vulgaris* ubiquitin-like protein mRNA, complete cds (U77939) | 07 | 98 | 7e-131 |
| CBFi45 | 256 | JZ468994 | E-CC/M-GTG | + | NC | *Oryza sativa* polyubiquitin (RUBQ1) gene,complete cds (AF184279) | 02 | 88 | 4e-76 |
| CBFi46 | 149 |  | E-CC/M-GTG | + | NC | Molecular chaperone (DnaJ superfamily) (Phvul.001G211800.1) | 01 | 100 | 9.6e-53 |
| CBFi55 | 234 |  | E-CT/M-GCT | NC | － | DEAD box containing helicases (Phvul.003G001700.1)_ | 03 | 100 | 2.9e-49 |
| CBFi66 | 130 | JZ469064 | E-TC/M-CGA | + | NC | Calreticulin (Phvul.008G052500.1) | 08 | 100 | 7.6e-47 |
| CBFi67 | 94 |  | E-CT/M-GCT | NC | + | 40S ribosomal protein S6 (Phvul.006G184300.1) | 06 | 100 | 1.3e-22 |
| CBFi71 | 168 | JZ469065 | E-AC/M-CAC | ++ | + | Protease inhibitor/seed storage/LTP family (Phvul.007G058500.1) | 07 | 100 | 1.4e-57 |
| CBFi83 | 216 | JZ469010 | E-CG/M-CAT | + | － | *Nelumbo nucifera* small molecular heat shock protein 10 (HSP10) mRNA, complete cds (EF421193) | 01 | 78 | 2e-34 |
| CBFi84 | 83 | JZ469068 | E-CG/M-CCA | ++ | NC | Alpha/Beta hydrolase fold-containing protein (Phvul.002G226600.1) | 02 | 100 | 2.3e-12 |
| CBFi97 | 141 | JZ469013 | E-TC/M-CGA | + | NC | *Phaseolus vulgaris* cultivar Sanilac eukaryotic translation initiation factor 4E (eIF4E) mRNA, partial cds (EF571271) | 06 | 100 | 6e-51 |
| CBFi98 | 301 | JZ469073 | E-TG/M-CGA | + | NC | Translation initiation factor 4F, cap-binding subunit (eIF-4E) and related cap-binding proteins (Phvul.007G270900.1) | 07 | 100 | 1e-119 |
| CBFi108 | 105 | JZ469075 | E-TC/M-GCA | ++ | NC | 40S ribosomal protein S19 (Phvul.005G018600.1) | 05 | 100 | 4.7e-10 |
| CBFi111 | 147 | JZ469021 | E-TT/M-CTC | + | － | *Pisum sativum* DEAD box RNA helicase mRNA, complete cds (AY167670) | 09 | 88 | 4e-34 |
| CBFi132 | 151 | JZ469031 | E-AT/M-CAT | + | NC | PREDICTED: *Oryctolagus cuniculus* ribosomal protein S6-like (LOC100339973), mRNA (XM002710901) | 08 | 78 | 3e-17 |
| CBFi144 | 89 |  | E-CA/M-CGT | + | NC | Class II aminotransferase/8-amino-7-oxononanoate synthase (Phvul.001G024100.1) | 01 | 100 | 2.9e-24 |
| CBFi170 | 207 | JZ469053 | E-TGC/M-CT | + | NC | *Ricinus communis* ubiquitin-protein ligase, putative, mRNA (XM002524909) | 07 | 80 | 2e-08 |
| CBFi172 | 238 | JZ469055 | E-TG/M-CGG | － | +++ | *Glycine max* mRNA for 14-3-3 protein, partial (AJ004898) | 08 | 97 | 2e-106 |
| **Development and cytoskeletal organization** | | | | | | | | | |
| CBFi2 | 218 |  | E-AA/M-TA | ++ | + | Nucleoporin-related (Phvul.001G145600.1) | 01 | 100 | 2.9e-93 |
| CBFi9 | 262 | JZ468985 | E-TG/M-AT | +++ | + | *Ricinus communis* non-symbiotic hemoglobin, putative, mRNA (XM002519062 ) | 11 | 85 | 1e-52 |
| CBFi14 | 142 |  | E-GC/M-TA | ++ | + | No apical meristem (NAM) protein (Phvul.001G176100.1) | 01 | 100 | 3.8e-51 |
| CBFi52 | 107 | JZ468995 | E-TC/M-GCA | － | NC | *Penaeus monodon* clone AF-E53M34-3 sex and growth trait AFLP marker genomic sequence (DQ087274) | 03 | 84 | 4e-08 |
| CBFi59 | 94 | JZ469061 | E-AA/M-CAA | + | NC | GRAS domain family protein (Phvul.010G129400.1) | 10 | 100 | 3.1e-24 |
| CBFi102 | 214 | JZ469017 | E-AC/M-CCA | ++ | + | PREDICTED: *Apis mellifera* tubulin alpha-1 chain-like (LOC550827), mRNA (XM623217) | 07 | 87 | 1e-56 |
| CBFi105 | 97 | JZ469018 | E-CA/M-CCT | ++ | + | *Nicotiana benthamiana* myosin Ⅷ-2 mRNA, complete cds (DQ875139) | 05 | 79 | 5e-05 |
| CBFi110 | 94 | JZ469076 | E-TT/M-CTC | ++ | + | Minichromosome maintenance protein 10 (Phvul.009G119500.1) | 09 | 100 | 7.4e-26 |
| CBFi125 | 94 | JZ469081 | E-CT/M-GGC | + | NC | SWI/SNF-related chromatin binding protein (Phvul.010G001800.1) | 10 | 100 | 1.3e-22 |
| CBFi136 | 77 | JZ469082 | E-AT/M-GCG | ++ | NC | Late embryogenesis abundant protein (Phvul.002G326000.1) | 02 | 100 | 2.2e-18 |
| CBFi139 | 140 | JZ469084 | E-TC/M-GCG | NC | + | Hat dimerisation domain-containing protein (Phvul.002G225800.1) | 02 | 100 | 9.5e-2 |
| CBFi145 | 108 | JZ469037 | E-TC/M-GGA | + | NC | *Glycine max* syntaxin mRNA, complete cds (AF532627) | 03 | 96 | 3e-28 |
| **Hormone responses** | | | | | | | | | |
| CBFi28 | 292 | JZ468987 | E-CG/M-CC | +++ | + | *Glycine max* GH3 gene for auxin-regulated protein (X60033) | 03 | 80 | 6e-46 |
| CBFi162 | 155 |  | E-TC/M-GTC | NC | － | Thyroid hormone receptor-associated coactivator complex component (TRAP170) (Phvul.006G101900.1) | 06 | 100 | 3.1e-59 |
| **Defense and stress responses** | | | | | | | | | |
| CBFi31 | 123 | JZ468988 | E-CC/M-CG | － | +++ | *Phaseolus vulgaris* mRNA (pR-1) for glutamine synthetase (GS, EC 6.3.1.2) (X04001) | 02 | 100 | 6e-07 |
| CBFi96 | 73 | JZ469012 | E-GC/M-CAT | + | NC | *Phaseolus vulgaris* mRNA (pR-2) for glutamine synthetase (GS,EC 6.3.1.2) (X04002) | 04 | 97 | 2e-13 |
| CBFi171 | 147 | JZ469054 | E-ACT/M-TT | ++ | NC | *Phaseolus vulgaris* NBS-LRR-like (*Co-2*) pseudogene, partial sequence (AF098969.1) | 11 | 100 | 3e-68 |
| **Signal transduction** | | | | | | | | | |
| CBFi7 | 106 |  | E-AT/M-GG | NC | +++ | Plant calmodulin-binding protein (Phvul.005G031900.1) | 05 | 100 | 1.8e-34 |
| CBFi34 | 198 |  | E-AA/M-GGT | +++ | NC | Calcium binding protein (Phvul.001G102700.1) | 01 | 100 | 4.4e-84 |
| CBFi41 | 145 |  | E-CT/M-GTG | + | NC | Leucine-rich repeat receptor-like protein kinase (Phvul.005G036600.1) | 05 | 100 | 1.8e-55 |
| CBFi42 | 196 | JZ468991 | E-CC/M-GAA | + | － | *Glycine max* seed calcium dependent protein kinase a mRNA, complete cds (AY247754) | 02 | 96 | 1e-51 |
| CBFi48 | 146 |  | E-CC/M-GGA | NC | － | Acetyltransferase (Phvul.002G044100.1) | 02 | 100 | 5.2e-56 |
| CBFi58 | 187 | JZ469000 | E-CT/M-CGT | +++ | NC | *Lotus japonicus* mRNA for calcium-binding protein (cbp1 gene) (AJ251808) | 11 | 76 | 2e-15 |
| CBFi60 | 106 | JZ469001 | E-AT/M-GCC | + | NC | Transcriptional repressors of the hairy/E(spl) family (contains HLH) (Phvul.007G206000.1) | 07 | 100 | 7.6e-33 |
| CBFi61 | 184 | JZ469062 | E-AT/M-GCG | + | － | F-box protein FBX9 (Phvul.009G127300.1) | 09 | 100 | 7.3e-43 |
| CBFi63 | 196 | JZ469002 | E-AA/M-CAG | + | NC | *Ricinus communis* protein phosphatase 2c, putative, mRNA (XM002525162) | 07 | 89 | 3e-18 |
| CBFi99 | 192 | JZ469014 | E-CG/M-CTT | + | － | PCDC2 (programmed cell death protein 2)-related (Phvul.009G166400.1) | 09 | 100 | 3.2e-79 |
| CBFi104 | 199 |  | EGA/M-CGG | + | － | Senescence regulator (Phvul.007G220200.1) | 07 | 100 | 6.5e-82 |
| CBFi106 | 89 | JZ469074 | E-CA/M-CGT | NC | + | MAPKK/MEKK/Serine-threonine protein kinase FUSED (Serine-threonine protein kinase FUSED) | 06 | 100 | 1e-23 |
| CBFi109 | 158 | JZ469020 | E-TG/M-CAA | + | NC | *Phaseolus vulgaris* serine-threonine kinase (AAR13300) | 08 | 100 | 1e-60 |
| CBFi116 | 73 | JZ469022 | E-GT/M-GCG | NC | + | Homeobox transcription factor, putative (Phvul.002G232200.1) | 02 | 100 | 1.6e-13 |
| CBFi122 | 287 | JZ469025 | E-GA/M-CAT | + | － | *Arabidopsis thaliana* RAN1 (RESPONSIVE-TO-ANTAGONIST 1); ATPase, coupled to transmembrane movement of ions, phosphorylative mechanism / copper ion t> (RAN1) mRNA, complete cds (NM123847) | 09 | 78 | 1e-51 |
| CBFi134 | 70 | JZ469032 | E-AT/M-CAT | + | NC | *Lotus japonicus* mRNA for Ser/Thr protein kinase, complete cds, clone: LNZ404 (AB184975) | 07 | 92 | 7e-07 |
| CBFi140 | 174 | JZ469035 | E-TC/M-GCC | + | － | *Ricinus communis* signalosome subunit, putative, mRNA (XM_002529164) | 05 | 85 | 4e-36 |
| CBFi143 | 81 |  | E-TC/M-GGC | + | NC | PH domain-containing protein (Phvul.011G177600.1) | 11 | 100 | 2e-19 |
| CBFi147 | 136 | JZ469039 | E-TG/M-GAT | + | NC | *Gossypium hirsutum* MAP kinase-like protein mRNA, complete cds (AY207316) | 08 | 76 | 2e-06 |
| CBFi155 | 85 | JZ469044 | E-TC/M-GCT | ++ | NC | *Glycine max* clone cw64 receptor-like kinase mRNA, complete cds (FJ014746) | 06 | 83 | 8e-08 |
| CBFi160 | 116 | JZ469047 | E-CG/M-CGG | NC | + | *Glycine max* NAC2 protein (NAC2) gene, complete cds (AY974350) | 01 | 97 | 2e-36 |
| **Gene expression and RNA metabolism** | | | | | | | | | |
| CBFi18 | 99 |  | E-CC/M-TA | NC | + | Zinc finger protein with krab and scan domains (Phvul.009G089000.1) | 09 | 100 | 1e-30 |
| CBFi56 | 178 | JZ468998 | E-CG/M-CTG | +++ | + | *Phaseolus vulgaris* PvGAI2 mRNA for DELLA protein, complete cds (AB304458) | 01 | 100 | 2e-71 |
| CBFi81 | 124 | JZ469009 | E-CT/M-GCT | + | NC | *Medicago truncatula* zinc finger protein, GH1 protein (EU306659) | 02 | 76 | 9e-10 |
| CBFi87 | 96 | JZ469011 | E-GA/M-GCT | NC | + | Apoptosis-promoting RNA-binding protein TIA-1/TIAR (RRM superfamily) (Phvul.001G019600.1) | 01 | 100 | 3.5e-30 |
| **Energy metabolism** | | | | | | | | | |
| CBFi10 | 90 |  | E-TG/M-TA | + | NC | AMP-binding enzyme (Phvul.003G010600.1) | 03 | 100 | 2.4e-25 |
| CBFi150 | 92 | JZ469086 | E-AT/M-GCC | ++ | + | ATP-citrate lyase (Phvul.002G064600.1) | 02 | 100 | 1.3e-22 |
| CBFi156 | 129 | JZ469045 | E-TC/M-CGA | + | NC | *Ricinus communis* ATP binding protein, putative, mRNA (XM002520023) | 11 | 81 | 2e-18 |
| **Redox reactions** | | | | | | | | | |
| CBFi76 | 217 | JZ469006 | E-AG/M-CGC | + | － | *Manihot esculenta* secretory peroxidase PX3 mRNA, complete cds (AY973612) | 03 | 78 | 4e-31 |
| CBFi78 | 105 | JZ469007 | E-TC/M-CGA | + | NC | Oxidoreductase, 2OG-Fe(II) oxygenase family protein (Phvul.004G120700.1) | 04 | 100 | 4.4e-23 |
| CBFi121 | 185 | JZ469024 | E-GG/M-CAG | + | NC | *Glycine max* glutathione S-transferase GST 23 mRNA, complete cds (AF243378) | 02 | 88 | 2e-45 |
| CBFi151 | 75 | JZ469087 | E-TG/M-CGG | NC | + | Aldo/keto reductase (Phvul.001G113300.1) | 01 | 100 | 1.6e-13 |
| **Protein Transport** | | | | | | | | | |
| CBFi12 | 238 |  | E-GT/M-TG | NC | + | Synaptic vesicle transporter SVOP and related transporters (major facilitator superfamily) (Phvul.009G189000.1) | 09 | 100 | 5.8e-90 |
| CBFi35 | 134 |  | E-TA/M-GTC | NC | + | Protein binding/protein transporter/structural molecule (Phvul.010G002800.1) | 10 | 100 | 1.5e-49 |
| CBFi75 | 74 | JZ469005 | E-CA/M-CGA | ++ | + | Predicted transporter (major facilitator superfamily) (Phvul.006G095300.1) | 06 | 100 | 2.5e-17 |
| CBFi94 | 76 | JZ469072 | E-CA/M-GCT | + | － | Transmembrane amino acid transporter protein (Phvul.008G283000.1) | 08 | 100 | 2.2e-18 |
| CBFi152 | 166 | JZ469088 | E-AG/M-CGC | + | － | Amino acid transporters (Phvul.002G235600.1) | 02 | 100 | 3.6e-65 |
| CBFi163 | 87 | JZ469089 | E-TC/M-GGC | － | NC | Sodium-dependent phosphate transporters (Phvul.011G016300.1) | 11 | 100 | 1.5e-21 |
| CBFi167 | 131 | JZ469051 | E-TG/M-GGT | + | NC | Sugar transporter/spinster transmembrane protein (Phvul.002G138900.1) | 02 | 100 | 6.3e-48 |
| **Unknown** | | | | | | | | | |
| CBFi8 | 261 | JZ469056 | E-TG/M-AT | ++ | + | Soybean clone JCVI-FLGm-13A8 unknown mRNA (BT098904) | 02 | 75 | 3e-14 |
| CBFi15 | 70 |  | E-GC/M-TC | NC | + | Kelch repeat domain protein (Phvul.009G169100.1) | 09 | 100 | 3.4e-15 |
| CBFi40 | 127 |  | E-CT/M-GAC | NC | + | Protein of unknown function (DUF1677) (Phvul.007G058200.1) | 07 | 100 | 3.1e-45 |
| CBFi86 | 88 | JZ469069 | E-TG/M-CGC | NC | ++ | Soybean clone JCVI-FLGm-20K19 unknown mRNA (BT095966) | 03 | 92 | 6e-16 |
| CBFi92 | 154 | JZ469071 | E-GA/M-GCC | + | － | *Naegleria gruberi* predicted protein, mRNA (XM002680880) | 03 | 75 | 4e-15 |
| CBFi113 | 69 | JZ469077 | E-TT/M-GCC | ++ | － | Soybean clone JCVI-FLGm-19L1 unknown mRNA (BT095609) | 07 | 90 | 2e-07 |
| CBFi120 | 87 | JZ469079 | E-GG/M-CAT | NC | + | Plant protein of unknown function (Phvul.009G240500.1) | 09 | 100 | 2.8e-24 |
| CBFi133 | 140 |  | E-AT/M-CAT | + | － | *Arabidopsis* proteins of unknown function (Phvul.002G073600.1) | 02 | 100 | 8.9e-53 |
| CBFi138 | 142 | JZ469083 | E-AT/M-GCC | NC | + | Soybean clone JCVI-FLGm-14G7 unknown mRNA (BT093355) | 02 | 92 | 2e-39 |
| CBFi141 | 85 | JZ469085 | E-TC/M-GCT | + | NC | Protein of unknown function, DUF573 (Phvul.004G052300.1) | 04 | 100 | 1.7e-20 |
| **No hit** | | | | | | | | | |
| CBFi91 | 89 | JZ469070 | E-CG/M-CCA | + | NC | No significant similarity found | 10 | — | — |
| CBFi101 | 99 | JZ469016 | E-TG/M-CCA | NC | ++ | No significant similarity found | 07 | — | — |
| CBFi117 | 77 | JZ469023 | E-GG/M-GCG | NC | ++ | No significant similarity found | 06 | — | — |
| CBFi123 | 63 | JZ469080 | E-GA/M-CAA | + | NC | No significant similarity found | 08 | — | — |
| CBFi135 | 83 | JZ469033 | E-AT/M-CAT | + | NC | No significant similarity found | 01 | — | — |
| CBFi146 | 163 | JZ469038 | E-TG/M-GTA | + | NC | No significant similarity found | 04 | — | — |
| CBFi168 | 105 | JZ469090 | E-GT/M-GCT | ++ | NC | No significant similarity found | 01 | — | — |
| CBFi3 | 128 |  | E-AA/M-TA | +++ | ++ | No significant similarity found | 02 | — | — |
| CBFi17 | 297 |  | E-CC/M-AC | NC | + | No significant similarity found | 06 | — | — |
| CBFi20 | 147 |  | E-CC/M-TG | +++ | NC | No significant similarity found | 08 | — | — |
| CBFi29 | 93 |  | E-CG/M-CC | + | NC | No significant similarity found | 08 | — | — |
| CBFi62 | 87 |  | E-AG/M-CTC | + | NC | No significant similarity found | 09 | — | — |
| CBFi159 | 104 |  | E-CT/M-CCT | ++ | NC | No significant similarity found | 11 | — | — |
| CBFi164 | 143 |  | E-TG/M-GAT | + | NC | No significant similarity found | 04 | — | — |

^a^Genbank entry at NCBI (http://www.ncbi.nlm.nih.gov/genbank/)

^b^Symbols: +++, ++, and + stand for strongly induced, + Transcripts >2 folds; ++ Transcripts > 2 folds and < 5 folds; +++ Transcripts >10 folds, NC stand for no change, while – refers to total suppression

^c^The TDF sequences were aligned and identified in Genbank database at NCBI or common bean reference genome database at Phytozome website

^d^Based on the Blast and hits (E value > e -5) with annotated ESTs
