# Supplementary material for: Differentially Expressed Genes in Resistant and Susceptible Common Bean (Phaseolus vulgaris L.) Genotypes in Response to Fusarium oxysporum f. sp. phaseoli
Source: PLoS One. 2015 Jun 1;10(6):e0127698. doi: 10.1371/journal.pone.0127698 (PMC4452237; doi:10.1371/journal.pone.0127698)
Supplement: S2 Table — (DOC) [file pone.0127698.s004.doc]

Table S2. Primers used for 19 target cDNA clones for real time quantitative polymerase chain reaction analysis (qRT-PCR) in *F. oxysporum* f. sp. *phaseoli* infected susceptible BRB130 and resistant CAAS260205 common bean genotypes.

| Target cDNA | Forward primer (5’–3’) | Reverse primer (5’–3’) | Product size (bp) |
| --- | --- | --- | --- |
| CBFi28 | GAACCCTGTGACTTGGAGGA | GCCTTTAGTCGACCTTGCTG | 115 |
| CBFi43 | CCTTGACGTTGTCAATGGTG | CCAAGATCCAGGACAAGGAA | 228 |
| CBFi45 | CAATTCCCTCCTTGTCCTGA | AGGAGTCCACCCTTCACCTT | 150 |
| CBFi54 | CGTTTGAAAGGAGACATCGG | ATTCCTCTGAGTGCCCCTGT | 112 |
| CBFi56 | CTGTGTCCATGACCTTGTCG | GAACATGCTCGAGATCCGTC | 107 |
| CBFi57 | GGTGGATGGATTGAAGTGCT | CGTACCAATTCATGGAGCAA | 116 |
| CBFi58 | ACGTCTTCGATCAGAACCGC | GTCCGCTTTCACGATCATCT | 125 |
| CBFi63 | GAGTCCTGAGTAACAGCCTCTTGGT | ATCAACATCATCCCACACACTCCAT | 104 |
| CBFi72 | CACCTATTGGCCGAAACTGT | CTCCCGGAGTACAGAAACCA | 101 |
| CBFi76 | CGCTCTGGCTACTAGGGATG | CCAATTCAGGGTGAGGAAGA | 128 |
| CBFi83 | CCTCAGTTCCCCCATACTCA | AGTTATTGCTGTTGGCCCTG | 107 |
| CBFi97 | CTGAGTAACGACGACGACGA | GCGTACCAATTCTCCAGAGG | 128 |
|  |  |  |  |
|  |  |  |  |
| CBFi109 | GTGTTGGAGGCAACCTTTGT | TCTCAGCAACAGCATCCAAC | 147 |
| CBFi111 | CAAGTGCCCGCATAACTTTC | GCGTACCAATTCTTCAGCAA | 111 |
| CBFi121 | TATGCAGATCGAATGTCCCA | CGAGTGCTGACGTTTGGATA | 108 |
| CBFi122 | CATAGTTGCAATGGTGGGTG | CACATAGTCTGCGGCTTCAA | 115 |
|  |  |  |  |
| CBFi170 | TCTGCGGCAACTCTATTC | GTAACTGCTCCAGAACGAC | 73 |
| CBFi171 | ATGGTTGCCTTCACTTGGAC | GGATGCAAAAGCAGAAGAGC | 118 |
| CBFi172 | CGTCAGATCGGGCTTGTAAT | CGCCTATTCACCTGGTTGTT | 206 |
| Actin | GAAGTTCTCTTCCAACCATCC | TTTCCTTGCTCATTCTGTCCG | 175 |
